# ﻿Phylogenetic relationships, distribution, and conservation of Roosmalens’ dwarf porcupine, *Coendouroosmalenorum* Voss & da Silva, 2001 (Rodentia, Erethizontidae)

**DOI:** 10.3897/zookeys.1179.108766

**Published:** 2023-09-11

**Authors:** Fernando Heberson Menezes, Thiago Borges Fernandes Semedo, Juliane Saldanha, Guilherme Siniciato Terra Garbino, Hugo Fernandes-Ferreira, Pedro Cordeiro-Estrela, Itayguara Ribeiro da Costa

**Affiliations:** 1 Programa de Pós-Graduação em Sistemática, Uso e Conservação da Biodiversidade, Departamento de Biologia, Centro de Ciências, Campus do Pici, Universidade Federal do Ceará, Fortaleza, Ceará, 60455-760, Brazil; 2 Laboratório de Mamíferos, Departamento de Sistemática e Ecologia, Centro de Ciências Exatas e da Natureza, Campus I, Universidade Federal da Paraíba, Castelo Branco, João Pessoa, Paraíba, 58051-900, Brazil; 3 CIBIO, Centro de Investigação em Biodiversidade e Recursos Genéticos, InBIO Laboratório Associado, Campus de Vairão, Universidade do Porto, 4485-661 Vairão, Portugal; 4 BIOPOLIS Program in Genomics, Biodiversity and Land Planning, CIBIO, Campus de Vairão, 4485-661 Vairão, Portugal; 5 Departamento de Biologia, Faculdade de Ciências, Universidade do Porto, 4099-002 Porto, Portugal; 6 Laboratório de Mastozoologia, Instituto de Biociências, Universidade Federal de Mato Grosso, Av. Fernando Corrêa da Costa, 2367, Cuiabá, Mato Grosso 78060-900, Brazil; 7 Museu de Zoologia João Moojen, Departamento de Biologia Animal, Universidade Federal de Viçosa, Viçosa, Minas Gerais, 36570-900, Brazil; 8 Laboratório de Conservação de Vertebrados Terrestres (Converte), Universidade Estadual do Ceará, Quixadá, Ceará, 63900-000, Brazil; 9 Seteg Soluções Geológicas e Ambientais, Fortaleza, Ceará, 60130-420, Brazil

**Keywords:** Amazonia, Brazil, cytochrome *b*, Madeira Province, Neotropical porcupines

## Abstract

The New World porcupines of the genus *Coendou* comprise 16 species of arboreal nocturnal rodents. Some of these species are poorly known and have not been included in phylogenetic analyses. Based on recently collected specimens with associated tissue from the Brazilian Amazonia, we investigate the distribution and phylogenetic relationships of Roosmalens’ dwarf porcupine, *Coendouroosmalenorum*, using an integrative approach using mitochondrial gene sequences and morphological data from new specimens and localities. Our results recovered *C.roosmalenorum* in the subgenus Caaporamys. However, analyses of our molecular and combined datasets produced different topologies. The new record shows the presence of *C.roosmalenorum* 480 km to the southeast of the Rio Madeira and 95 km away from Rio Juruena in Mato Grosso state, indicating a wider distribution in southern Amazonia than suspected. All known records of *C.roosmalenorum* are in the Madeira biogeographical province, to which it might be endemic.

## ﻿Introduction

New World porcupines of the genus *Coendou* Lácepède, 1799 are arboreal, herbivorous, nocturnal rodents of the family Erethizontidae which occur in tropical and subtropical regions of the Americas (Eisenberg and Redford 1999). Erethizontids are characterized mainly by having their fur modified into quills, and some species have a bulbous snout and a long dorsally prehensile tail, which is an exclusive trait among prehensile-tailed mammals (Emmons 1997; Voss 2015). Currently the 18 species of erethizontids are classified in three genera, with the genus *Coendou* comprising 16 species (Menezes et al. 2021).

Since the early 1990s, studies have clarified the taxonomic status and phylogenetic relationships among species of *Coendou* (Handley and Pine 1992; Voss and Angermann 1997; Voss and da Silva 2001; Voss 2011; Feijó and Langguth 2013; Mendes Pontes et al. 2013; Voss et al. 2013), as well as its subgeneric classification (e.g., Menezes et al. 2021). Additionally, knowledge of the distribution and natural history of *Coendou* species has advanced (e.g., Freitas et al. 2013; Gregory et al. 2015; Menezes et al. 2020; Ramírez-Chaves et al. 2020a).

Most current knowledge about species of *Coendou* is restricted to taxa that occur close to urban centers (see Voss 2015). In Amazonia, most records are from the margins of the main rivers. Neotropical porcupines are usually hard to observe because they are not captured by the usual live-trapping sampling methods and have cryptic behavior (Kays and Allison 2001; Gregory et al. 2015; Kaizer et al. 2022). Nevertheless, *Coendou* species can be locally abundant in some areas, such as *Coendoumelanurus* (Wagner, 1842) in French Guiana (Vié 1999). Hence, information on species distributed in the Amazonian rainforest or along the Andean foothills is scarce. Of the 16 *Coendou* species that have been assessed by the International Union for the Conservation of Nature (IUCN), *Coendousperatus*Mendes Pontes et al. 2013 is classified as Endangered (Roach and Mendes Pontes 2020) and six are classified as Data Deficient. Among the latter group is Roosmalens’ dwarf porcupine, *C.roosmalenorum* Voss & da Silva, 2001 (Delgado 2016a, 2016b; Roach 2016; Roach and Naylor 2016; Weksler et al. 2016a, 2016b).

*Coendouroosmalenorum* is one of the least known New World species of porcupine. It was originally thought to occur only in the Brazilian Amazon from both banks of the Madeira River, and only anecdotal information is available on its natural history (Voss and da Silva 2001). Since the description of the species, no novel data have been published on its biology, natural history, or distribution. An exception is a Menezes et al.’s (2021) morphological phylogeny which included *C.roosmalenorum* in the subgenus Caaporamys Menezes, Feijó, Fernandes-Ferreira, da Costa & Cordeiro-Estrela, 2021. Due to the scarcity of information and the lack of fresh tissue samples, the phylogenetic relationships of this species remains uncertain. Herein, we investigate these topics based on a recently collected specimen with associated tissue, from Aripuanã, Mato Grosso, western Brazil.

## ﻿Materials and methods

### ﻿Specimens examined

**Figure 1.**
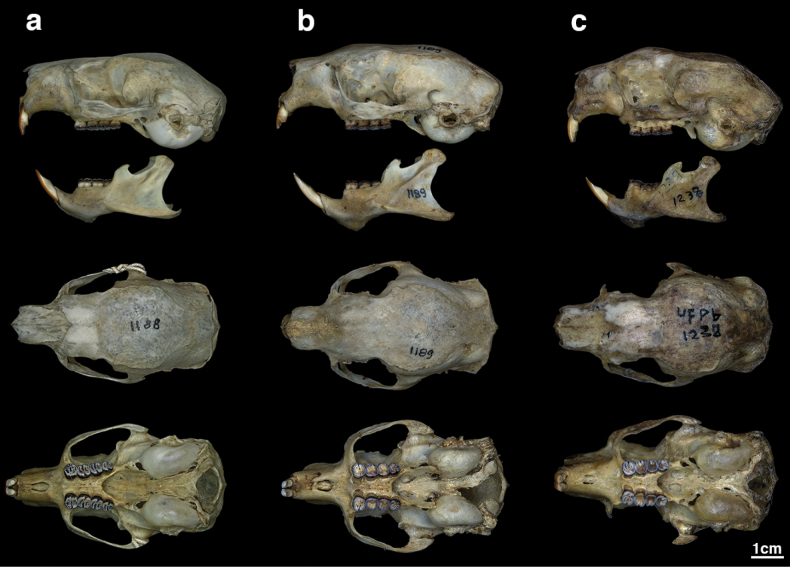
Skulls and mandibles of *Coendouroosmalenorum* specimens deposited in Coleção de Mamíferos da Universidade Federal da Paraíba (UFPB) **a**UFPB 1188 **b**UFPB 1189 **c**UFPB 1238.

**Figure 2.**
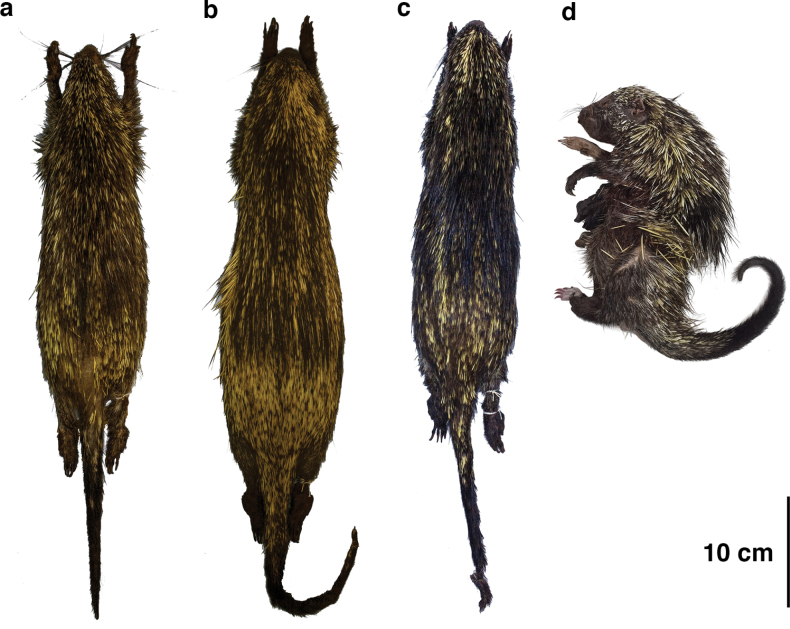
Stuffed skins (**a–c**) and entire fluid-preserved specimen (**d**) of *Coendouroosmalenorum***a**UFPB 1188 **b**UFPB 1189 **c**UFPB 1238 **d**UFMT 4930.

The examined specimens are deposited in two collections (abbreviations in parentheses): Coleção de Mamíferos da Universidade Federal de Mato Grosso (UFMT), Cuiabá and Coleção de Mamíferos da Universidade Federal da Paraíba (UFPB), João Pessoa (Figs 1, 2). The holotype and paratype of *Coendouroosmalenorum* are deposited in the scientific collection of the Instituto Nacional de Pesquisas da Amazônia (INPA), Manaus (Voss and da Silva 2001). The recently collected specimen (UFMT 4930) from which we obtained the tissue sample was accidentally killed by a bulldozer during a forest suppression activity at a mining site in Serra do Expedito, Aripuanã, Mato Grosso (10°40'S, 59°30'W). It is preserved in fluid, and a muscle sample was obtained and preserved in 70% ethanol. The skull is severely damaged but external characters of the pelage and quills allowed us to identify the specimen as *C.roosmalenorum*.

The nomenclature of the soft hairs and quills of the porcupines used here follows Menezes et al. (2021). Skull characters follow Menezes et al. (2021) and the descriptions of *C.roosmalenorum* by Voss and da Silva (2001) and Voss (2015).

### ﻿DNA purification and sequencing

Genomic DNA of UFMT 4930 was obtained using a saline extraction protocol (Aljanabi and Martinez 1997). We used the primers MVZ05 and MVZ16 (Smith and Patton 1993) to amplify a fragment of the mitochondrial cytochrome *b* gene (cyt *b*), following the protocol described by Saldanha et al. (2019). Purification and sequencing were obtained by the Sanger method in both directions with the same amplification primers on an ABI3730xl Genetic Analyzer at the Biotecnologia, Pesquisa e Inovação, São Paulo, Brazil. We obtained a partial cyt *b* sequence with 821 nucleotide length. This sequence has been deposited in GenBank with accession number OR400787.

### ﻿Phylogenetic analyses

To investigate the phylogenetic position of *Coendouroosmalenorum*, we analyzed a molecular dataset and a dataset that combined molecular sequences with morphological character data.

Only cyt *b* sequences were available for our molecular analysis; these and our new sequence of *C.roosmalenorum* augmented the molecular dataset previously published by Menezes et al. (2021) (Suppl. material 4). The dataset had a total of 60 sequences, which were aligned using the MUSCLE algorithm in MEGA 11 (Tamura et al. 2021). We did not trim the final alignment and treated the ends of partial sequences as missing data, resulting in an alignment size of 1140 nucleotides. Before the phylogenetic analyses, we tested the best substitution model using the ModelFinder plugin (Kalyaanamoorthy et al. 2017) in the PhyloSuite environment (Zhang et al. 2020). The best-fit model selected according to the Bayesian information criterion (BIC) (see Sullivan and Joyce 2005) was HKY (Hasegawa et al. 1985) with gamma distribution (+G). The HKY+G model was used in all phylogenetic analyses. After model selection, we ran a maximum-likelihood (ML) and Bayesian-inference (BI1) analyses using *Chaetomyssubspinosus* (Olfers, 1818) and *Erethizondorsatum* (Linnaeus, 1758) as outgroups. The single available sequence of *Coendoupruinosus* Thomas, 1905 was not included because its cyt *b* sequence is short with only 248 nucleotides (KC463880).

An ML consensus tree was inferred using the IQ-TREE (Nguyen et al. 2015) plugin for the PhyloSuite environment under 5000 ultrafast (Minh et al. 2013) bootstraps, as well as the Shimodaira–Hasegawa-like approximate likelihood-ratio test (Guindon et al. 2010), an initial BioNJ tree method (Guindon and Gascuel 2003), and four categories of gamma distribution (gamma = 0.258). Estimated nucleotide frequencies are f(A) = 0.304, f(C) = 0.265 f(G) = 0.124, f(T) = 0.307. BI1 phylogenies were inferred using the MrBayes 3.2.6 (Ronquist et al. 2012) plugin for the PhyloSuite environment with eight chains over 10 million generations sampled every 100, in which the initial 25000 sampled data were discarded as burn-in to estimate consensus trees and evolutionary parameter.

Our combined dataset included the same cyt *b* sequences described above and the morphological character matrix of Menezes et al. (2021) to produce a nexus file following the procedure described by Maddison et al. (1997). To detect the autapomorphies of *C.roosmalenorum*, we performed a maximum-parsimony (MP) analysis with 1000 bootstrap replicates in PAUP4 (Swofford 2002; Wilgenbusch and Swofford 2003). The starting tree(s) were obtained via stepwise addition and the tree-bisection-reconnection (TBR) algorithm with a reconnection limit of 8. Branches were collapsed (creating polytomies) if maximum branch lengths were zero. The consensus tree used the 50% majority-rule consensus rule. After the MP analysis, we ran BI2 by using partitioned models in the MrBayes plugin of the PhyloSuite environment. We used the same model and parameters of BI1 for the cyt *b* partition and the Mk model for discrete characters of Lewis (2001) for the morphological partition.

Genetic distances were calculated for the cyt *b* dataset in MEGA 11 (Tamura et al. 2021) using Kimura’s 2-parameter (K2P) model (Kimura 1980) considering no gamma distribution or invariant sites following previous studies (e.g., Mendes Pontes et al. 2013; Torres-Martínez et al. 2019). All ambiguous positions were removed for each sequence pair (pairwise deletion option).

### ﻿Distribution and conservation data

To provide an updated map of the geographic distribution of *C.roosmalenorum*, we used published records of museum specimens (Voss and da Silva 2001; Voss 2015), together with new records obtained by us (Table 1). Our map includes both geopolitical boundaries (South American countries and Brazilian states) and the biogeographical regions proposed by Morrone et al. (2022).

**Table 1.** List of the known localities of the Brazilian endemic *Coendouroosmalenorum*. Numbers in the “Locality” column refer to the map (Fig. 5).

	Locality	GPS coordinates	Reference
1	Serra do Expedito, Aripuanã, Mato Grosso	10°40'S, 59°30'W	This study
2	Samuel Hydroelectric Dam, Rio Jamari, Rondônia	8°45'S, 63°27'W	This study
3	BR 364, 49 km E from Porto Velho, Rondônia*	8°45'S, 63°30'W	Voss and da Silva 2001
4	Novo Jerusalém on Lago Matupirizinho, Amazonas	5°33'S, 61°07'W	Voss and da Silva 2001
5	Santa Maria on Lago Matupiri, Amazonas	5°33'S, 61°15'W	Voss and da Silva 2001

*Coordinates were estimated in this study.

We calculated the Extent of Occurrence (EOO), which is the area contained within the smallest continuous boundary that can be drawn to encompass all known, inferred, or projected points of the current presence of a taxon (IUCN 2012). Based on IUCN recommendations, we estimated the EOO using the minimum convex polygon (Burgman and Fox 2003). The Area of Occupancy (AOO) is defined as the area inside the EOO occupied by the species (IUCN 2012). Because *C.roosmalenorum* is an arboreal mammal, we assumed as the AOO the forest cover currently available based on Projeto Mapbiomas (Souza et al. 2020). This same database was used to estimate the deforestation in the polygon between 1987 and 2020. These analyzes were performed using QGIS v. 3.32.

## ﻿Results

**Figure 3.**
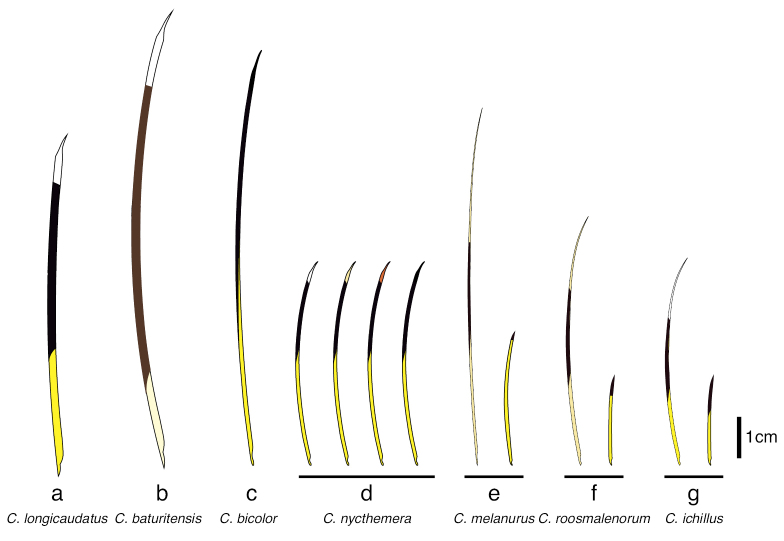
Quills and bristle-quills of select Brazilian porcupine species (*Coendou* spp.) **a** Amazonian *C.longicaudatus*, long tricolored quill **b***C.baturitensis*, long tricolored quill **c***C.bicolor*, long bicolored quill **d***C.nycthemera*, long quills with different distal band colors **e***C.melanurus*, tricolored guard hair and bicolored quill **f***C.roosmalenorum*, tricolored bristle-quill and bicolored quill **g***C.ichillus*, tricolored bristle-quill and bicolored quill. Adapted from Menezes et al. (2020).

The specimens we examined all have the diagnostic characters of *Coendouroosmalenorum* (Fig. 3): brownish dorsal fur covering the quills; bristle-quills with strong yellowish B1, blackish B2, and light yellowish B3 on the dorsal crest; bicolored short quills with long yellowish B1 and very short blackish B2; blackish, unicolored bristles on tail; small body size when compared to other porcupine species; no nasofrontal inflation on the skull; and tail length subequal to body length (Voss and da Silva 2001; Menezes et al. 2020).

Our MP analysis of the combined (molecular + morphological) dataset resulted in the following indices: tree length = 1206 steps, consistency index (CI) = 0.4934, homoplasy index (HI) = 0.5066, CI excluding uninformative characters = 0.4337, HI excluding uninformative characters = 0.5663, retention index (RI) = 0.8231, rescaled consistency index (RC) = 0.4061, f value = 53305, f-ratio = 0.3722.

**Figure 4.**
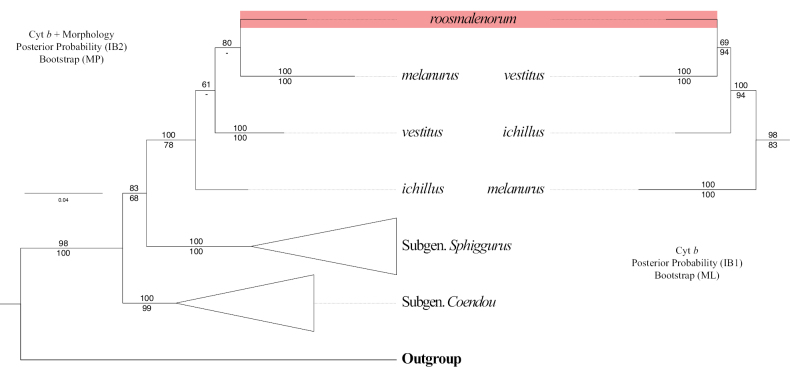
Phylogenetic hypotheses of Coendou focused on the species of subgenus Caaporamys based on two distinct datasets. Left: combined morphological and cyt *b* datasets, the values above the branches represent the posterior probabilities of IB2 and values below represent the bootstrap supports of MP. Right: cyt *b*; values above the branches represent the posterior probabilities of IB1, and values below represent the bootstrap proportions of ML. Branches of subgenera other than *Caaporamys* are collapsed.

Both datasets recovered *C.roosmalenorum* in the subgenus Caaporamys, as expected based on morphological traits. However, the molecular dataset (ML and BI1) and combined dataset (MP and BI2) had two distinct topologies (Fig. 4). The molecular dataset recovered *C.roosmalenorum* as the sister taxon of *C.vestitus*, while the combined dataset suggests that *C.roosmalenorum* is the sister species of *C.melanurus*. The internal relationships among the four *Caaporamys* species differ using both datasets. Additionally, the species with smallest genetic distance from *C.roosmalenorum* is *C.vestitus* (Table 2).

**Table 2.** Average pairwise K2P sequence distances (scaled as percentages) at the cyt *b* locus among species of subgenus Caaporamys.

	*C.ichillus*	*C.melanurus*	*C.roosmalenorum*
*C.melanurus*	8.2%		
*C.roosmalenorum*	5.3%	7.8%	
*C.vestitus*	5.7%	8.4%	5.1%

Our MP analysis of the combined data revealed a single morphological apomorphy in *C.roosmalenorum*, which is a weakly developed lambdoidal ridge (Char25:1, CI 0.4, Table 3). There is no morphological synapomorphy for the *C.melanurus* + *C.roosmalenorum* hypothesis, as the MP analysis resulted in a polytomy for relationships among species of *Caaporamys*.

**Table 3.** List of morphological apomorphies of *Caaporamys* species obtained in maximum parsimony of combined data.

Subgenus Caaporamys	Character	Steps	CI	State change	Description
*C.ichillus*	Char1	1	0.250	1 → 0	Fur not covering quills on the dorsal crest
	Char2	1	0.500	1 → 0	Absence of dorsal fur
	Char11	1	0.500	2 → 0	B3 of bristle-quills is whitish
	Char21	1	0.400	0 → 1	Medial masseter scar oval and wide
	Char24	1	0.667	0 → 1	Temporal crests drawing in dorsal view V-shaped
	Char26	1	0.333	0 → 1	Palatal keel conspicuous
	Char31	1	0.200	1 → 0	Dorsal roof of the external auditory meatus not keeled
	Char33	1	0.500	1 → 0	Orbitotemporal fossa shallow
*C.melanurus*	Char12	1	0.429	0 → 1	B2 about the same length of B1
	Char13	1	0.500	2 → 1	B3 about the same length of B2
*C.vestitus*					(No morphological apomorphies)
*C.roosmalenorum*	Char25	1	0.400	0 → 1	Lambdoidal ridge weakly developed

**Figure 5.**
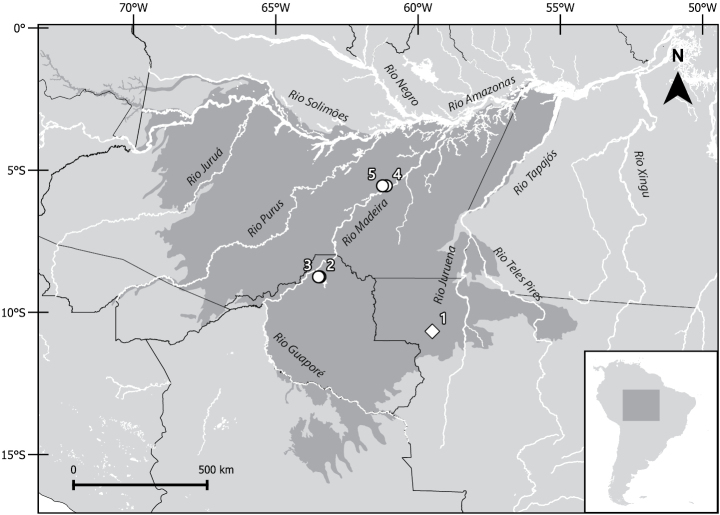
Distribution of *Caaporamysroosmalenorum* in Brazilian Amazonia. Data for the numbered localities are provided in Table 1. The new record (locality 1) is the southeastern most record for the species, from Mato Grosso state, Brazil. The darker gray area represents the Madeira Province sensu Morrone et al. (2022).

All the known records of *C.roosmalenorum* are from the Madeira Province of Amazonia (Fig. 5). The new record (locality 1) is 480 km east of the Samuel Hydroelectric Dam (localities 2 and 3) and 590 km south of the Matupiri lake region (localities 4 and 5). The EOO is estimated to be 108,050 km^2^, and the AOO is 107,764 km^2^.

## ﻿Discussion

### ﻿Phylogenetic relationships of *Coendouroosmalenorum*

The molecular and combined datasets recovered *Coendouroosmalenorum* as a member of the subgenus Caaporamys, as expected from its morphological characters. This species has the diagnostic morphological traits of the subgenus, such as the presence of bristle-quills in the dorsal pelage, soft ventral pelage, and unicolored caudal bristles on the tail (Menezes et al. 2021). The new information we obtained agrees with the single previous phylogeny that included the species (Menezes et al. 2021), which reinforces the importance of external characters for the subgeneric classification of *Coendou*.

However, the two datasets analyzed in this report suggest different topologies, with *C.roosmalenorum* placed either as the sister species of *C.melanurus* or *C.vestitus*. These differing results may be due to the unavailability of morphological data for *Caaporamys* species. The available morphological dataset for phylogenetic analyses lacks cranial characters for *C.melanurus* and includes only molecular characters for *C.vestitus*. In this way, the very homoplasic characters as colors and length of the quills bands appear to have a major contribution to the combined data in the absence of cranial characters.

The color and length of quill bands of Neotropical porcupines have a high level of homoplasy (Table 3; Menezes et al. 2021). Homoplasy is known to have negative effects on phylogenetic inference, such as the reduction of branch supports, artificial grouping (Simpson 2010; Radel et al. 2013), and long-branch attraction (Bergsten 2005). Therefore, we understand the topology of the combined dataset as a case of grouping by homoplasy and consider *C.vestitus* as the sister species of *C.roosmalenorum* until new data are obtained.

### ﻿Distribution and conservation of *C.roosmalenorum*

Previously, the distribution of *C.roosmalenorum* was associated with the Rio Madeira, as the species was known from only two localities along that river or its tributary, Rio Jamari (Voss and da Silva 2001). The new record shows the presence of *C.roosmalenorum* 480 km to the southeast of the Rio Madeira and 95 km away from Rio Juruena in Mato Grosso state, indicating a wider distribution in southern Amazonia, as suspected (Voss 2015). The new record comes from the Amazon rainforest region, which is not subject to seasonal flooding.

All the known records of *C.roosmalenorum* are in the Madeira Province sensu Morrone et al. (2022). Therefore, we consider the possibility of this porcupine species occurs only in the Madeira Province, or is even more restricted, as are other arboreal mammal species such as the following primates: *Callicebuscinerascens*, *C.ornatus*, *C.stephennashi*, *C.brunneus*, *C.cupreus* (see the map in Carneiro et al. 2016), *Micohumeralifer*, *M.humilis*, *M.chrysoleucos*, *M.marcai*, *M.rondoni* (Garbino 2014; Garbino and Nascimento 2014), and *Chiropotesalbinasus* (Ferrari et al. 1999). The distribution of *C.roosmalenorum* may be delimited in the northwest by the Rio Madeira, in the southwest by the Rio Guaporé, and in the east by the Rio Juruena.

Distribution patterns suggest that *Coendou* species can occur in sympatry with other porcupine subgenera and are only allopatric with species of the same subgenus. *Coendouroosmalenorum* occurs in sympatry with *C.longicaudatus* Daudin, 1802, the largest porcupine species of the subgenus Coendou, and it is allopatric with other *Caaporamys* species. *Coendouichillus* Voss & da Silva 2001 is the *Caaporamys* species that occurs closest to *C.roosmalenorum*, with records north of the Solimões and Amazonas rivers (Menezes et al. 2020) and west of the Madeira and Guaporé rivers (Gregory et al. 2015). A similar pattern occurs with *C.nycthemera* (Olfers, 1818), of the subgenus Sphiggurus F. Cuvier, 1823, which is sympatric with the larger *C.baturitensis* Feijó & Langguth, 2013 throughout most of its distribution and is allopatric with other *Sphiggurus* species, such as *C.bicolor* (Tschudi, 1844) and *C.speratus*Mendes Pontes et al. 2013 (Freitas et al. 2013; Leal et al. 2017; Menezes et al. 2020), which are the closely related to *C.nycthemera* (Mendes Pontes et al. 2013; Menezes et al. 2021). Also species of the subgenus Coendou are allopatric but sympatric with species of other subgenera; *C.prehensilis* (Linnaeus 1758) is sympatric with *C.speratus* (Menezes et al. 2021) and *C.rufescens* is sympatric with *Coendouichillus* (Ramírez-Chaves et al. 2016).

*Coendouroosmalenorum* is classified as Data Deficient by the IUCN (Roach and Naylor 2016) due the absence of recent information on its status and ecological requirements. The EOO and AOO here documented are certainly underestimated because of the sampling gaps but much larger than threshold for Vulnerable (IUCN 2012). However, this polygon has lost 9.34% of forest cover since 1987, almost completely replaced by pasture (9.13%) (Souza et al. 2020). Considering the Madeira Province, most of its territory is in the Brazilian states of Amazonas and Rondônia, which have lost approximately 15,400 and 15,500 km^2^ of forest cover, respectively, between 2008 and 2022. The municipality of Aripuanã (Mato Grosso state), from where the new record of *C.roosmalenorum* came, has lost 1,226 km^2^ of forest cover in the same period (INPE 2023). In addition to habitat loss, *Coendou* species face other threats in South America such as subsistence hunting, predation by domestic dogs, and roadkills (Ramírez-Chaves et al. 2020b, 2021). Moreover, a recent infection by Brazilian porcupine poxvirus has been described (Hora et al. 2021) and recorded in two *Coendou* species of different subgenera: C. (Sphiggurus) spinosus (F. Cuvier, 1823) (Guerra et al. 2022) and Coendou (Coendou) longicaudatus (Silva et al. 2023).

Therefore, it is necessary to investigate population size and decline to determine the conservation status of *C.roosmalenorum* more accurately following IUCN criteria. In addition to standard ecological approaches focused on erethizontids, such as visual census, telemetry, and arboreal camera traps (e.g., Giné et al. 2015; Bowler et al. 2017; Melo-Dias et al. 2023), we suggest that alternative sampling methods may yield new records of this poorly known species. Thermal drones, for example, have been efficient to survey and monitor large primates and could be used for other arboreal mammals (de Melo 2021). Citizen-science data, mainly animals photographed by the community, may provide new records, as attested by a recent publication on the genus by Ramírez-Chaves et al. (2020b).

## ﻿Additional information

### Author ORCIDs

Fernando Heberson Menezes https://orcid.org/0000-0003-4169-0215

Thiago Borges Fernandes Semedo https://orcid.org/0000-0003-4379-5993

Juliane Saldanha https://orcid.org/0000-0003-3983-7169

Guilherme Siniciato Terra Garbino https://orcid.org/0000-0003-1701-5930

Hugo Fernandes-Ferreira https://orcid.org/0000-0001-9064-3736

Pedro Cordeiro-Estrela https://orcid.org/0000-0003-3383-571X

Itayguara Ribeiro da Costa https://orcid.org/0000-0003-0135-6706

### Data availability

All of the data that support the findings of this study are available in the main text or Supplementary Information.

## Supplementary materials

Supplementary material 1 Dataset 1 Data type nex Explanation note Nexus file of aligned cytochrome *b* sequences of Erethizontid used for ML and IB1. https://binary.pensoft.net/file/903991This dataset is made available under the Open Database License (http://opendatacommons.org/licenses/odbl/1.0/). The Open Database License (ODbL) is a license agreement intended to allow users to freely share, modify, and use this Dataset while maintaining this same freedom for others, provided that the original source and author(s) are credited.Fernando Heberson Menezes, Thiago Borges Fernandes Semedo, Juliane Saldanha, Guilherme Siniciato Terra Garbino, Hugo Fernandes-Ferreira, Pedro Cordeiro-Estrela, Itayguara Ribeiro da Costa

Supplementary material 2 Dataset 2 Data type nex Explanation note Nexus file of aligned cytochrome *b* and morphological data of Erethizontid used for MP. https://binary.pensoft.net/file/903992This dataset is made available under the Open Database License (http://opendatacommons.org/licenses/odbl/1.0/). The Open Database License (ODbL) is a license agreement intended to allow users to freely share, modify, and use this Dataset while maintaining this same freedom for others, provided that the original source and author(s) are credited.Fernando Heberson Menezes, Thiago Borges Fernandes Semedo, Juliane Saldanha, Guilherme Siniciato Terra Garbino, Hugo Fernandes-Ferreira, Pedro Cordeiro-Estrela, Itayguara Ribeiro da Costa

Supplementary material 3 Dataset 3 Data type nex Explanation note Nexus file of aligned cytochrome *b* and morphological data of Erethizontid used for IB2. https://binary.pensoft.net/file/903993This dataset is made available under the Open Database License (http://opendatacommons.org/licenses/odbl/1.0/). The Open Database License (ODbL) is a license agreement intended to allow users to freely share, modify, and use this Dataset while maintaining this same freedom for others, provided that the original source and author(s) are credited.Fernando Heberson Menezes, Thiago Borges Fernandes Semedo, Juliane Saldanha, Guilherme Siniciato Terra Garbino, Hugo Fernandes-Ferreira, Pedro Cordeiro-Estrela, Itayguara Ribeiro da Costa

Supplementary material 4 Erethizontid cytochrome *b* sequences utilized in phylogenetic analyses Data type docx https://binary.pensoft.net/file/903994This dataset is made available under the Open Database License (http://opendatacommons.org/licenses/odbl/1.0/). The Open Database License (ODbL) is a license agreement intended to allow users to freely share, modify, and use this Dataset while maintaining this same freedom for others, provided that the original source and author(s) are credited.Fernando Heberson Menezes, Thiago Borges Fernandes Semedo, Juliane Saldanha, Guilherme Siniciato Terra Garbino, Hugo Fernandes-Ferreira, Pedro Cordeiro-Estrela, Itayguara Ribeiro da Costa
