# Supplementary material for: ﻿Phylogenetic relationships, distribution, and conservation of Roosmalens’ dwarf porcupine, Coendouroosmalenorum Voss & da Silva, 2001 (Rodentia, Erethizontidae)
Source: Zookeys. 2023 Sep 11;1179:139–55. doi: 10.3897/zookeys.1179.108766 (PMC10507447; doi:10.3897/zookeys.1179.108766)
Supplement: Supplementary material 4 — Erethizontid cytochrome b sequences utilized in phylogenetic analyses [file zookeys-1179-139_article-108766__-s004.docx]

| **Table S1:** Erethizontid cytochrome *b* sequences utilized in phylogenetic analyses. | | | | |  | |
| --- | --- | --- | --- | --- | --- | --- |
| **GenBank Access** | **Species** | **Voucher** | **Locality** | **Source** |  | |
| KY784123 | *Coendou baturitensis* | UFPB 9291 | Mulungu, Ceará, Brazil | Menezes *et al.,* 2021 |  | |
| KY784124 | *Coendou baturitensis* | UFPB 9780 | Mulungu, Ceará, Brazil | Menezes *et al.,* 2021 |  | |
| KY784125 | *Coendou baturitensis* | UFPB 9781 | Mulungu, Ceará, Brazil | Menezes *et al.,* 2021 |  | |
| KC463857 | *Coendou bicolor* | AMNH 214612 | Rio Mamoré, Beni, Bolivia | Voss *et al.,* 2013 |  | |
| KC463858 | *Coendou bicolor* | KU 144560 | Cajamarca, Peru | Voss *et al.,* 2013 |  | |
| KC463859 | *Coendou bicolor* | MUSM 9398 | Madre de Dios, Peru | Voss *et al.,* 2013 |  | |
| KC463860 | *Coendou bicolor* | FMNH 203679 | San Martím, Peru | Voss *et al.,* 2013 |  | |
| KC463861 | *Coendou ichillus* | TTU 115491 | Loreto, Peru | Voss *et al.,* 2013 |  | |
| KC261591 | *Coendou insidiosus* | UFES 136 | Nova Viçosa, Bahia, Brazil | Mendes Pontes *et al.*, 2013 |  | |
| AF411581 | *Coendou longicaudatus boliviensis* | UnB 1757 (Manso 212) | UHE Manso, Mato Grosso, Brazil | Bonvicino *et al.,* 2002 |  | |
| AF411582 | *Coendou longicaudatus boliviensis* | UnB 1758 (Manso 138) | UHE Manso, Mato Grosso, Brazil | Bonvicino *et al.,* 2002 |  | |
| AF411584 | *Coendou longicaudatus boliviensis* | Manso 849 | UHE Manso, Mato Grosso, Brazil | Bonvicino *et al.,* 2002 |  | |
| KC463873 | *Coendou longicaudatus boliviensis* | AMNH 262274 | San Ramon, Bolivia | Voss *et al.,* 2013 |  | |
| U34851 | *Coendou longicaudatus longicaudatus* | INPA 3919 (MNFS 1016) | Fazenda Santa Fé, Acre, Brazil | Lara *et al.*, 1996 |  | |
| U34852 | *Coendou longicaudatus longicaudatus* | MVZ 195088 (MNFS 439) | Eirunepé, Amazonas, Brazil | Lara *et al.,* 1996 |  | |
| KC463866 | *Coendou longicaudatus longicaudatus* | USNM 560869 | Cerro Neblina Base Camp, Amazonas, Venezuela | Voss *et al.,* 2013 |  | |
| KC463867 | *Coendou longicaudatus longicaudatus* | AMNH 273130 | Nuevo San Juan, Loreto, Peru | Voss *et al.,* 2013 |  | |
| KC463868 | *Coendou longicaudatus longicaudatus* | MUSM 15324 | Nuevo San Juan, Loreto, Peru | Voss *et al.,* 2013 |  | |
| KC463869 | *Coendou longicaudatus longicaudatus* | MVZ 155200 | Huampami, Amazonas, Peru | Voss *et al.,* 2013 |  | |
| KC463870 | *Coendou longicaudatus longicaudatus* | MVZ 155201 | Huampami, Amazonas, Peru | Voss *et al.,* 2013 |  | |
| KC463871 | *Coendou longicaudatus longicaudatus* | MVZ 191349 | Rio Juruá, Ocidente, Acre, Brazil | Voss *et al.,* 2013 |  | |
| KC463872 | *Coendou longicaudatus longicaudatus* | INPA 2875 | Eirunepé, Amazonas, Brazil | Voss *et al.,* 2013 |  | |
| KC463874 | *Coendou longicaudatus longicaudatus* | MNHN 1997.643 | Petit Saut, French Guiana | Voss *et al.,* 2013 |  | |
| KC463875 | *Coendou longicaudatus longicaudatus* | EBRG 23415 | Río Caroní, Bolívar, Venezuela | Voss *et al.,* 2013 |  | |
| KC463876 | *Coendou longicaudatus longicaudatus* | USNM 281898 | Valledupar, Cesar, Colombia | Voss *et al.,* 2013 |  | |
| KC463877 | *Coendou longicaudatus longicaudatus* | USNM 281904 | Valledupar, Cesar, Colombia | Voss *et al.*, 2013 |  | |
| KC463878 | *Coendou longicaudatus longicaudatus* | USNM 443409 | Las Mesas, Táchira, Venezuela | Voss *et al.,* 2013 |  | |
| KC463879 | *Coendou longicaudatus longicaudatus* | USNM 528360 | Limoncocha, Sucumbíos, Ecuador | Voss *et al.,* 2013 |  | |
| MG775435 | *Coendou longicaudatus longicaudatus* | IAvH 123 | Toledo, Norte de Santander, Colombia | Torres-Martínez *et al.*, 2019 |  | |
| AF411583 | *Coendou melanurus* | UFPB 3001 | São João da Baliza, Roraima, Brazil | Bonvicino *et al.,* 2002 |  | |
| KC463862 | *Coendou melanurus* | MNHN 1997.641 | Barragem Petit Saut, French Guiana | Voss *et al.,* 2013 |  | |
| KC463863 | *Coendou mexicanus* | ASNHC 6407 | Campeche, Mexico | Voss *et al.,* 2013 |  | |
| KC261597 | *Coendou nycthemera* | UFES 2079 | UHE Estreito, Tocantins, Brazil | Mendes Pontes *et al.,* 2013 |  | |
| KC463864 | *Coendou nycthemera* | USNM 519690 | Ilha de Marajó, Pará, Brazil | Voss *et al.,* 2013 |  | |
| KC463865 | *Coendou nycthemera* | USNM 519692 | Ilha de Marajó, Pará, Brazil | Voss *et al.,* 2013 |  | |
| KY784126 | *Coendou prehensilis* | UFPB 9412 | João Pessoa, Paraíba, Brazil | This work |  | |
| HM462243 | *Coendou prehensilis* | MNRJ 73383* | Usina Trapiche, Pernambuco, Brazil | Leite *et al.*, 2011 |  | |
| KC463880 | *Coendou pruinosus* | MHNLS 7692*** | Zulia, Venezuela | Voss *et al.,* 2013 |  | |
| KC463881 | *Coendou quichua* | KMH 2218 | Cotopaxi, Ecuador | Voss *et al.,* 2013 |  | |
| KC463882 | *Coendou quichua* | LACM 27376 | Cesar, Colombia | Voss *et al.,* 2013 |  | |
| KC463883 | *Coendou quichua* | USNM 296308 | Zona Canal, Panama | Voss *et al.,* 2013 |  | |
| KC463884 | *Coendou rufescens* | AMNH 181483 | Cauca, Colombia | Voss *et al.,* 2013 |  | |
| OR400787 | *Coendou roosmalenorum* | UFMT 4930 | Aripuanã, Mato Grosso, Brazil | In this work |  | |
| KC261592 | *Coendou speratus* | UFPE 1708 | Usina Trapiche, Pernambuco, Brazil | Mendes Pontes *et al.,* 2013 |  | |
| KC261593 | *Coendou speratus* | UFPE 1709 | Usina Trapiche, Pernambuco, Brazil | Mendes Pontes *et al.,* 2013 |  | |
| KC261594 | *Coendou speratus* | MNRJ 72046 | Usina Trapiche, Pernambuco, Brazil | Mendes Pontes *et al.,* 2013 |  | |
| KC261595 | *Coendou speratus* | MNRJ 72045** | Usina Trapiche, Pernambuco, Brazil | Mendes Pontes *et al.,* 2013 |  | |
| KC261596 | *Coendou speratus* | UFES 1184 | Usina Trapiche, Pernambuco, Brazil | Mendes Pontes *et al.,* 2013 |  | |
| KC463885 | *Coendou spinosus* | UNMZ (GD 252) | Itapúa, Paraguay | Voss *et al.,* 2013 |  | |
| KC463886 | *Coendou spinosus* | UMMZ 174975 | Caazapá, Paraguay | Voss *et al.,* 2013 |  | |
| KC463887 | *Coendou spinosus* | UFMG 3043 | Sorocaba, São Paulo, Brazil | Voss *et al.,* 2013 |  | |
| AF407277 | *Coendou spinosus* | MNRJ 46938 | Rio das Ostras, Rio de Janeiro, Brazil | Bonvicino *et al.,* 2002 |  | |
| AF411580 | *Coendou spinosus* | MNRJ 46937 | Sumidouro, Rio de Janeiro, Brazil | Bonvicino *et al.,* 2002 |  | |
| EU544661 | *Coendou spinosus* | CIT1326 | UHE Rosal, Espírito Santo, Brazil | Vilela *et al.*, 2009 |  | |
| EU544662 | *Coendou spinosus* | MZUSP 35142 | Biritiba Mirim, São Paulo, Brazil | Vilela *et al.,* 2009 |  | |
| JX312693 | *Coendou spinosus* | MNRJ 78937 | Santa Tereza, Espírito Santo, Brazil | Voloch *et al.,* 2013 |  | |
| KC463888 | *Coendou vestitus* | AMNH 70596 | Cundinamarca, Colombia | Voss *et al.,* 2013 |  | |
| MG383643 | *Coendou vestitus* | IAvH 7956 | Colombia, Villa de Leyva | Ramírez-Chaves *et al.*, 2019 |  | |
| FJ357428 | *Erethizon dorsatum* | – | – | Vilela *et al.,* 2009 |  | |
| KC463889 | *Erethizon dorsatum* | USNM 568658 | – | Voss *et al.,* 2013 |  | |
| EU544660 | *Chaetomys subspinosus* | MCNU 918 | Salvador, Bahia, Brazil | Vilela *et al.,* 2009 |  | |
| * Neotype of *Coendou prehensilis*  ** Holotype of *Coendou speratus*  *** We avoided to use the sequence of *Coendou pruinosus* since it is a short fragment | | | | | |  |

**References of cytochrome *b* sequences**

[Bonvicino CR, Penna-Firme V, Braggio E (2002) Molecular and Karyologic Evidence of the Taxonomic Status of *Coendou* and *Sphiggurus* (Rodentia: Hystricognathi). Journal of Mammalogy 83: 1071–1076.](https://sciwheel.com/work/bibliography/13898627)

[Lara MC, Patton JL, da Silva MNF (1996) The simultaneous diversification of South American echimyid rodents (Hystricognathi) based on complete cytochrome b sequences. Molecular Phylogenetics and Evolution 5: 403–413. https://doi.org/10.1006/mpev.1996.0035](https://sciwheel.com/work/bibliography/13898841)

[Leite YLR, Caldara Jr V, Loss AC, Costa LP, Melo ÉRA, Gadelha JR, Mendes Pontes AR (2011) Designation of a neotype for the Brazilian porcupine, *Coendou prehensilis* (Linnaeus, 1758). Zootaxa 2791: 30–40.](https://sciwheel.com/work/bibliography/13898634)

[Mendes Pontes AR, Gadelha JR, Melo ÉRA, de Sá FB, Loss AC, Caldara Junior V, Costa LP, Leite YLR (2013) A new species of porcupine, genus *Coendou* (Rodentia: Erethizontidae) from the Atlantic forest of northeastern Brazil. Zootaxa 3636: 421–438. https://doi.org/10.11646/zootaxa.3636.3.2](https://sciwheel.com/work/bibliography/13898638)

[Menezes FH, Feijó A, Fernandes‐Ferreira H, da Costa IR, Cordeiro‐Estrela P (2021) Integrative systematics of Neotropical porcupines of *Coendou prehensilis*](https://sciwheel.com/work/bibliography/13899410) complex (Rodentia: Erethizontidae). Journal of Zoological Systematics and Evolutionary Research 59: 2410–2439. https://doi.org/10.1111/jzs.12529

[Ramírez-Chaves HE, Torres-Martínez MM, Noguera-Urbano EA, Passos FC, Colmenares-Pinzón JE (2019) State of knowledge and potential distribution of the Colombian endemic brown hairy dwarf porcupine *Coendou vestitus* (Rodentia). Mammalian Biology 99: 1–11. https://doi.org/10.1016/j.mambio.2019.09.012](https://sciwheel.com/work/bibliography/13899401)

[Torres-Martínez MM, Ramírez-Chaves HE, Noguera-Urbano EA, Colmenares-Pinzón JE, Passos FC, García J (2019) On the distribution of the Brazilian porcupine *Coendou prehensilis* (Erethizontidae) in Colombia. Mammalia 83: 290–297. https://doi.org/10.1515/mammalia-2018-0043](https://sciwheel.com/work/bibliography/13899216)

[Vilela RV, Machado T, Ventura K, Fagundes V, de J Silva MJ, Yonenaga-Yassuda Y (2009) The taxonomic status of the endangered thin-spined porcupine, *Chaetomys subspinosus* (Olfers, 1818), based on molecular and karyologic data. BMC Evolutionary Biology 9: 29. https://doi.org/10.1186/1471-2148-9-29](https://sciwheel.com/work/bibliography/10914567)

[Voloch CM, Vilela JF, Loss-Oliveira L, Schrago CG (2013) Phylogeny and chronology of the major lineages of New World hystricognath rodents: insights on the biogeography of the Eocene/Oligocene arrival of mammals in South America. BMC Research Notes 6: 160. https://doi.org/10.1186/1756-0500-6-160](https://sciwheel.com/work/bibliography/4990495)

Voss RS, Hubbard C, Jansa SA (2013) Phylogenetic Relationships of New World Porcupines (Rodentia, Erethizontidae): Implications for Taxonomy, Morphological Evolution, and Biogeography. American Museum Novitates 3769: 1–36. https://doi.org/10.1206/3769.2
